# Neuromuscular Mechanisms Underlying Changes in Force Production during an Attentional Focus Task

**DOI:** 10.3390/brainsci10010033

**Published:** 2020-01-07

**Authors:** Shawn Wiseman, Shahab Alizadeh, Israel Halperin, Behzad Lahouti, Nicholas J. Snow, Kevin E. Power, Duane C. Button

**Affiliations:** 1 School of Human Kinetics and Recreation, Memorial University of Newfoundland, St. John’s, NL A1C 5S7, Canada; saw072@mun.ca (S.W.); salizadeh@mun.ca (S.A.); blahouti@mun.ca (B.L.); njsnow@mun.ca (N.J.S.); kevinp@mun.ca (K.E.P.); 2 School of Public Health, Sackler Faculty of Medicine, Tel-Aviv University, Tel-Aviv 6997801, Israel; ihalperin@tauex.tau.ac.il; 3 Sylvan Adams Sports Institute, Tel Aviv University, Tel-Aviv 6997801, Israel; 4 Faculty of Medicine, Memorial University of Newfoundland, St. John’s, NL A1C 5S7, Canada

**Keywords:** corticospinal excitability, attentional focus, co-contraction

## Abstract

We examined the effects of attentional focus cues on maximal voluntary force output of the elbow flexors and the underlying physiological mechanisms. Eleven males participated in two randomized experimental sessions. In each session, four randomized blocks of three maximal voluntary contractions (MVC) were performed. The blocks consisted of two externally and two internally attentional focus cued blocks. In one of the sessions, corticospinal excitability (CSE) was measured. During the stimulation session transcranial magnetic, transmastoid and Erb’s point stimulations were used to induce motor evoked potentials (MEPs), cervicomedullary MEP (CMEPs) and maximal muscle action potential (Mmax), respectively in the biceps brachii. Across both sessions forces were lower (*p* = 0.024) under the internal (282.4 ± 60.3 N) compared to the external condition (310.7 ± 11.3 N). Muscle co-activation was greater (*p* = 0.016) under the internal (26.3 ± 11.5%) compared with the external condition (21.5 ± 9.4%). There was no change in CSE. Across both sessions, force measurements were lower (*p* = 0.033) during the stimulation (279.0 ± 47.1 N) compared with the no-stimulation session (314.1 ± 57.5 N). In conclusion, external focus increased force, likely due to reduced co-activation. Stimulating the corticospinal pathway may confound attentional focus. The stimulations may distract participants from the cues and/or disrupt areas of the cortex responsible for attention and focus.

## 1. Introduction

The effects of attentional focus instructions on motor learning and performance have been extensively studied in the past 20 years [1,2]. Specifically, two types of instructions have been compared: those that elicit an internal focus (IF) and external focus (EF) of attention [1,2]. EF leads one to focus on the intended effects of movements on the environment. For example, focusing on the bull’s eye during a dart throwing task. Conversely, IF leads one to focus on a body part or muscle group. For example, focusing on wrist movement during a dart throwing task [3]. Many studies report that EF enhances motor learning and physical performance compared to IF [1,4,5,6,7]. This includes tasks that require accuracy, balance, strength and speed. The effects are consistent across children, adults, older adults, and those suffering from mental disease [8,9,10]. These effects are arguably some of the most established ones identified in human movement science.

Despite the impressive number of studies comparing attentional focus strategies across tasks and populations, little is known about the underpinning neuromuscular mechanisms that can explain the observed effects. A handful of studies examined if attentional focus strategies lead to different brain activation patterns using electroencephalography (EEG) and functional magnetic resonance imaging (fMRI) [11,12,13,14]. EEG alpha power is generally lower during EF and associated with more ideal alpha frequencies [15]. An fMRI study observed higher activation in the motor cortex during EF compared to IF. Thus, some evidence shows that various parts of the brain are activated differently between EF and IF which may account for enhanced neuromuscular performance during EF. The most commonly used tool to shed light on the mechanistic pathway explaining the superiority of EF is surface electromyography (EMG), which is a general measure of central nervous system excitation. A repeated—although not consistent—pattern is that EF leads to decreased muscle EMG activity from both the agonist and antagonist muscle groups involved in the task execution compared to IF [16]. The reduction in EMG activity during EF may promote effective and efficient movement patterns [17]. However, EEG, fMRI and EMG alone cannot pinpoint the motor pathways leading to the enhanced movement patterns associated with EF. Indeed, EF can promote superior motor performance by eliciting greater nervous system excitation, less inhibition, or a combination thereof from the brain to the spinal cord.

Nervous system excitation and inhibition can be examined through measuring corticospinal excitability via transcranial magnetic stimulation (TMS), and transmastoid electrical stimulation (TMES) [18,19]. TMS elicits a motor evoked potential (MEP) in a muscle of interest, while TMES elicits a cervicomedullary MEP. TMS-evoked MEP amplitudes are used to quantify corticospinal excitability (CSE) [20]. Alterations in CSE could occur anywhere along the corticospinal pathway (i.e., from cortex to motoneuron). The combined use of the aforementioned techniques is used to determine whether the modulation of CSE is predominantly supraspinal or spinal [18]. The corticospinal tract is examined due to its importance in the organization of single and multi-jointed movements. The corticospinal fibers control the spinal motoneurons that innervate the muscles of the trunk and limbs [21]. Many modulators have been shown to influence CSE from caffeine indigestion [22] to arousal imagery [23]. It is possible that EF may increase corticospinal excitability, decrease corticospinal inhibition, or a combination of both which would account, in part, for the increase in motor performance seen. This would further our understanding of the pathways and underlying mechanisms to address the changes in motor performance and learning seen with attentional focus feedback.

In view of the insight that can be gained using corticospinal excitability measurement techniques, and the limited knowledge accumulated to date on the pathways accounting for attentional focus instructions effects, combining the two in a single experiment is a worthwhile endeavor. Therefore, the aim of this study was to (1) compare CSE to the biceps brachii between EF and IF cued maximal voluntary contractions (MVC) of the elbow flexors and (2) compare co-activation patterns of the biceps brachii and triceps brachii between EF and IF cued MVC of the elbow flexors. We hypothesized that (1) CSE would be modulated differently between EF and IF cued condition and (2) co-activation would be greater with an IF cued condition.

## 2. Materials and Methods

Eleven resistance-trained males (1.77 ± 0.02 m, 84.32 ± 3.22 kg, 23.8 ± 2.36 years) participated in the experimental study. Resistance-trained status was determined as meeting the Canadian Society of Exercise Physiology guidelines of two hours a week of resistance training for at least a year. We chose to recruit only resistance-trained participants because corticospinal excitability is training dependent [24,25,26,27]. Participants completed a magnetic stimulation safety checklist prior to participation in order to screen for potential contraindications with magnetic stimulation procedures [28]. Verbal description about the procedures was given to the participants and if accepted they gave their informed written consent. The study was approved by The Memorial University of Newfoundland Interdisciplinary Committee on Ethics in Human Research and was in accordance with the Tri-Council guidelines in Canada with full disclosure of potential risks to participants (HK-20190008).

### 2.1. Elbow Flexor Force

Participants were seated in a custom-built chair (Technical Services, Memorial University of Newfoundland, St. John’s, NL, Canada) in an upright position, with chest and head strapped in place to minimize movement, with hips and knees flexed at 90°. The forearm was held horizontal, positioned supine with the shoulders resting against the back of the chair, and placed in a custom-made orthosis that was connected to a load cell (S-beam hanging load cells, model number LC101-500, Omegadyne, Inc., Sunbury, OH, USA) which was calibrated prior to the measurement. The load cell detected force output, which was amplified (×1000) (CED 1902, Cambridge Electronic Design Ltd., Cambridge, UK) and displayed on a computer screen. Data was sampled at 5000 Hz. Participants were instructed to maintain an upright position with their head in a neutral position during contractions. Visual feedback was given to all participants during all contractions prior to and during the conditions as a line on a computer screen in front of them showing when to begin and end the contraction. Participants were only able to view the amount of their force production and biceps brachii EMG activity.

### 2.2. Electromyography

Electromyography (EMG) activity was recorded from the dominant arm’s biceps brachii and triceps brachii using surface EMG (10 mm MediTrace Ag-AgCl, Graphic Controls Ltd., Buffalo, NY, USA). Electrodes were placed 2 cm apart (center to center) over the midpoint of the muscle belly of the participant’s biceps brachii and triceps brachii lateral head. A ground electrode was placed over the lateral epicondyle of the dominant knee. Skin preparation for all recording electrodes included shaving to remove excess hair and cleaning with an isopropyl alcohol swab to remove dry epithelial cells. An inter-electrode impedance of <5 kΩ was obtained prior to recording to ensure an adequate signal-to-noise ratio. EMG signals were amplified (×1000) (CED 1902) and filtered using a 3-pole Butterworth filter with cut-off frequencies of 10–1000 Hz. All signals were analog-digitally converted at a sampling rate of 5 kHz using a CED 1401 (Cambridge Electronic Design Ltd., Cambridge, UK) interface.

### 2.3. Stimulation Conditions

Motor responses from the bicep brachii were elicited via (1) transcranial magnetic stimulation (TMS), (2) transmastoid electrical stimulation (TMES) and (3) brachial plexus electrical stimulation at Erb’s point. Stimulation intensities used for TMS and TMES were adjusted similar to that of Pearcey et al. (2014) so that the evoked potentials produced by each, evoked motor evoked potentials (MEPs), and CMEPs, respectively, were of similar amplitude and normalized to a maximal M-wave (Mmax) [26]. Stimulation intensities were then set during an isometric elbow flexion contraction equal to 5% of MVC.

#### 2.3.1. Transcranial Magnetic Stimulation (TMS)

TMS- (MEPs) were used to measure corticospinal excitability. A TMS (Magstim 200, maximal output 2.0 Tesla) circular coil (13 cm outside diameter) was placed directly over the vertex of the head to induce MEPs in the active (5% MVC) biceps brachii muscle. The vertex was located by marking the measured halfway points between the nasion to inion and tragus to tragus. The coil was flipped to ensure the induced current flow was anterior to posterior in the target motor cortex (A side up for right side, B side up for left) to activate the dominant biceps brachii [29,30,31]. The optimum stimulation site was determined by initially placing the coil over the vertex and slightly moving the coil towards the non-dominant hemisphere in three directions, i.e., anteriorly, posteriorly, and laterally. Once the highest MEP response was elicited, that position was determined as the optimum stimulation site. Stimulation intensity was set to elicit a MEP 10%–20% of Mmax taken as an average of eight trials in the biceps brachii during a 5% MVC.

#### 2.3.2. Transmastoid Electrical Stimulation (TMES)

Stimulation (Model DS7AH, Digitimer Ltd., Welwyn Garden City, Hertfordshire, UK) was applied via surface electrodes placed over the mastoid processes and current was passed between them (200 µs duration, 80–200 mA). Stimulation intensity was adjusted to prevent ventral root activation by closely monitoring CMEP responses for any decrease in onset latency (~2 ms), which shows cervical ventral root activation [32]. Stimulation intensity was adjusted to elicit a response that matched the size of MEP amplitude, taken as an average of eight trials, in the biceps brachii during a 5% MVC.

#### 2.3.3. Brachial Plexus Stimulation

Stimulation (Model DS7AH, Digitimer Ltd., Welwyn Garden City, Hertfordshire, UK) of the brachial plexus was used to measure maximal compound muscle action potential (Mmax). Erb’s point was electrically stimulated via a cathode and anode placed on the skin over the supraclavicular fossa and the acromion process, respectively. Current pulses were delivered as a singlet (200 μs duration, 90–185 mA, 400 V). The electrical current was gradually increased until Mmax of the biceps brachii at a 5% MVC was observed.

### 2.4. Experimental Protocol

Participants completed a familiarization session and two experimental sessions that were randomized. Each session took place on separate days with 48 h between each session.

#### 2.4.1. Familiarization Session

Participants performed two 5 s MVCs of the dominant elbow flexors, with 2 min of rest between contractions. If the force from the two MVCs differed by greater than 5%, a third MVC was performed. Following completion of the MVCs, participants practiced holding the 5% MVC contraction for 10 s at each position. Participants then received the three different types of stimulations at various intensities to ensure that they were comfortable to endure the stimulation paradigm involved in each experimental session.

#### 2.4.2. Stimulation Session

**Figure 1:**
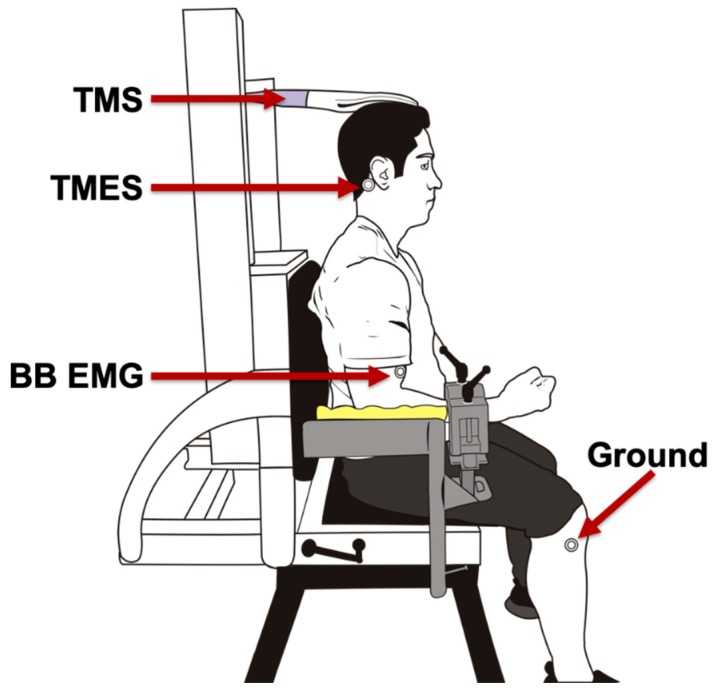
Participant’s experimental set-up. Participants were positioned up right in an elevated chair with shoulders at 0 degrees and elbows at 90 degrees. TMS: transcranial magnetic stimulation, TMES: transmastoid electrical stimulation, BB EMG: biceps brachii electromyography. Ground: ground electrode.

**Figure 2:**
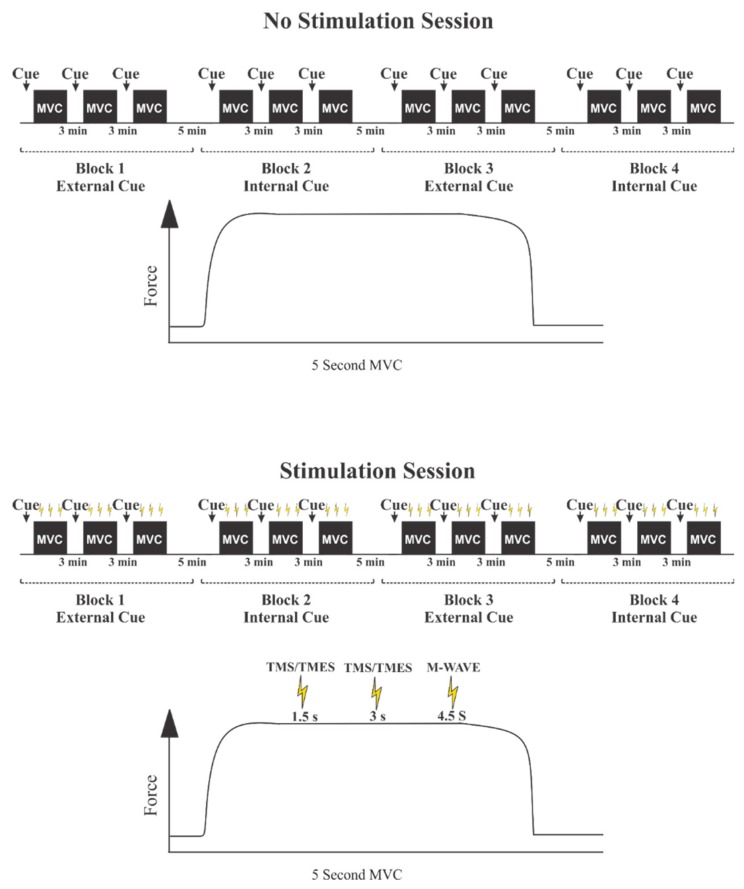
Experimental protocol. Each participant completed two experimental sessions (no stimulation session above, stimulation session below) which were randomized. Within each session, participants completed two blocks of three externally cued condition and two blocks of internally cued condition which were also randomized. Maximal voluntary contractions (MVC) were held for 5 s, beginning and ending at 2 and 7 s respectively, and during the stimulation session a transcranial magnetic stimulation (TMS) and transmastoid electrical stimulation (TMES) pulse was randomly delivered at 1.5 and 3.0 s marks with an M-Wave delivered each time at the 4.5 s mark. The traces underneath the sequence blocks represents the timing of the no-stimulation/stimulation during a 5 s MVC contraction.

Upon arrival, the participants were prepared for EMG and asked to perform two elbow flexor MVCs. A 10 min rest period was then issued to ensure no effect of the MVC on the CSE measurements [33]. Following the rest period, the experimental procedures began and the stimulation intensities for the Mmax, MEP, and CMEP of the biceps brachii during 5% MVC were determined. Participants then moved on to perform a semi-randomized protocol where they completed four blocks of 3 MVCs of the elbow flexors with 3 min of rest between MVCs. Five minutes of rest was given between each block of conditions. A total of 12 MVCs were performed. Participants were verbally encouraged with the same attentional focus cue provided immediately before each contraction in each block of conditions. Participants were either asked to “focus on pulling up on the handle as hard and as quickly as you possibly can” (external cue) or to “focus on contracting your biceps as hard and as quickly as you possibly can” (internal cue). In total participants were EF cued six times or IF cued six times. These cues were countered balanced between sets. During each condition participants received counter-balanced TMSs and TMESs at 1.5 and 3 s and an M-wave was given at the 4.5 s mark. See Figure 1 and Figure 2 for the experimental set-up.

#### 2.4.3. Non-Stimulation Session

If the stimulation session was completed first, then the non-stimulation session was completed 48 h after the first session. This session was identical to the stimulation session except no stimulations were used. This session was included in the study to examine if stimulations impact a participant’s ability to perform an MVC and their ability to focus on the attentional focus cues.

### 2.5. Data Analysis

Force, EMG, and CSE data were measured offline using Signal 4.0 software (Cambridge Electronic Design Ltd., Cambridge, Hertfordshire, UK). All offline computations were conducted using Microsoft Office Excel 2016 (Microsoft Corporation, Redmond, WA, USA).

**Maximum voluntary isometric force.** Peak elbow flexor’s force was obtained from all six MVCs under each condition (external cued condition and internal cued condition) during both no-stimulation and stimulation sessions. MVC force output was measured as the peak amplitude from no force to maximum force.

**Electromyography (EMG).** Root mean square EMG (rmsEMG) from the biceps brachii and triceps brachii muscles was calculated from *t* = 1 s to *t* = 2 s interval of each MVC once the force reached its peak output under each condition (external cued condition and internal cued condition) and during each session (no-stimulation and stimulation). Additionally, muscle co-activation was quantified by computing the percentage of triceps brachii rmsEMG/biceps brachii rmsEMG [34]. To illustrate the amount of co-activation per unit of force production and illustrate the relationship between force and coactivation, the percentage ratio of muscle co-activation per Newton of force was calculated for MVCs from both the external and internal cued conditions during both no-stimulation and stimulation sessions.

**Figure 3:**
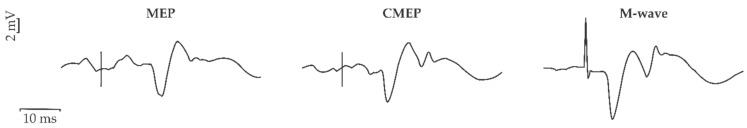
Raw data sample for MEP, CMEP and Mmax responses during an MVC from a single participant.

**Corticospinal excitability (CSE).** During the stimulation session only, biceps brachii MEP, CMEP, and Mmax peak-to-peak amplitudes (mV) were extracted during all six MVCs under each condition (external cued condition and internal cued condition). See Figure 3 for raw data for MEP, CMEP and Mmax responses during a MVC. Since amplitudes and areas give similar results, we used MEP, CMEP, and Mmax amplitudes for comparisons [35]. MEP and CMEP peak-to-peak amplitudes were normalized to Mmax amplitudes (%Mmax), given Mmax is a stable measure of muscle activity during maximal muscle fibre recruitment [36]. Simply put, the MEP and CMEP values were divided by the M-wave produced from the same trial. As well, ratios of normalized MEP/CMEP amplitude were calculated [37]. The MEP/CMEP would indicate the level of supraspinal or spinal excitability. Levels above 1 are indicative of higher supraspinal excitability compared to spinal excitability [30].

### 2.6. Statistical Analysis

Prior to statistical analyses all data underwent quality control checks in Microsoft Office Excel 2016 (Microsoft Corporation, Redmond, WA, USA) for missing data points and outliers. In terms of missing data, only one participant was unable to complete the stimulation session (P09). This participant was not included in CSE analyses; however, their MVC peak force and rmsEMG data (trial 1 to trial 6) for both the external condition and internal conditions (12 trials) were subsequently imputed for the stimulation session to enable groupwise comparisons across sessions. Additionally, two participants (P10, P11) were missing force data for one MVC trial each (trial 4), under both the internal and external conditions, for the stimulation session alone (four trials). The missing datapoints were in a time series where data for both sessions (i.e., stimulation and non-stimulation sessions) were not available. In total, 16 datapoints were missing for MVC peak force (6.1%) and 12 datapoints each were missing for rmsEMG of both biceps brachii (4.5%) and triceps brachii (4.5%). Missing data were imputed by determining the series average for the entire sample, including both conditions (external condition, internal condition), at their respective timepoints and sessions using the Missing Values Analysis and Transform functions in SPSS (V26.0, IBM Corporation, Armonk, NY, USA). Outliers were considered datapoints that exceeded the sample mean by ± three standard deviations (SD). No outliers were identified.

Statistical analyses were completed using SPSS (V26.0, IBM Corporation, Armonk, NY, USA). Assumptions of normality (Shapiro–Wilk test), sphericity (Mauchly’s test), and homogeneity of variances (Levene’s test) were tested for all outcome measures where appropriate. For the Shapiro–Wilk test, statistical significance was set at *p* < 0.001 [38]. In the event of a violation of the assumption of sphericity, *p*-values were adjusted using the Greenhouse–Geisser correction. If the assumption of homogeneity of variances was violated, *p*-values were adjusted (equal variances not assumed).

To rule out whether measures of MVC peak force, rmsEMG, or CSE changed over subsequent trials (trial 1 to trial 6), separate one-way repeated-measures analyses of variance (ANOVAs) with the factor TRIAL (6 levels) were conducted on all data independently for internal and external conditions, as well as stimulation and no-stimulation sessions. This test was used to guide subsequent analyses in terms of whether trials were pooled or tested separately. For MVC peak force, the main effect of TRIAL was statistically significant in all cases (*F*_(5, 50)_ ≥ 3.982, *p* ≤ 0.022). Similarly, with reference to rmsEMG data, the main effect of TRIAL was statistically significant in most cases (*F*_(5, 50)_ ≤ 6.690, *p* ≥ 0001). However, regarding CSE, the main effect of TRIAL was not statistically significant in any case (*F*_(5, 45)_ ≤ 2.137, *p* ≥ 0.150). Consequently, in main statistical tests, TRIAL was considered a separate factor for MVC peak force and rmsEMG data, whereas all levels of the factor TRIAL were pooled for CSE.

For main statistical tests, repeated-measures ANOVAs and paired-samples *t*-tests were used, with designs depending on the result of the above one-way repeated-measures ANOVAs. Peak force measurements from MVCs were compared across trials (trial 1 to trial 6), conditions (external cued condition and internal cued condition), and sessions (no-stimulation and stimulation) using a 6 × 2 × 2 three-way repeated-measures ANOVA with the factors TRIAL, CONDITION, and SESSION, respectively. Due to variability in EMG recordings, raw rmsEMG values for biceps brachii and triceps brachii were examined separately for each session (no-stimulation and stimulation) across trials (trial 1 to trial 6) and conditions (external cued condition and internal cued condition) using 2 × 2 two-way repeated measures ANOVAs with the factors TRIAL and CONDITION, respectively, given they were not normalized [33]. Because triceps brachii/biceps brachii co-activation values were normalized, they were compared as square root transformed values across trials (trial 1 to trial 6), conditions (external cued condition and internal cued condition), and sessions (no-stimulation and stimulation) using separate 6 × 2 × 2 three-way repeated measures ANOVAs with the factors TRIAL, CONDITION, and SESSION, respectively [33]. For CSE, average values across all trials (trial 1 to trial 6) for Mmax amplitude (mV), as well as MEP/Mmax, CMEP/Mmax, and square root transformed CMEP/MEP ratios, were compared across conditions (external cued condition and internal cued condition) using separate paired-samples *t*-tests. Finally, to investigate the relationship between changes in peak force and co-activation across stimulation conditions, two analyses were performed. First, a 2 × 2 two-way repeated-measures ANOVA with the factors CONDITION and SESSION was conducted on the percentage ratios of muscle co-activation per Newton of force calculated from MVCs from both the external and internal conditions during both no-stimulation and stimulation sessions. Last, simple bivariate correlations (Pearson’s *r*) were calculated between changes in MVC peak force and triceps brachii/biceps brachii co-activation from external to internal cued condition in the no-stimulation and stimulation sessions separately. Strength of the correlation coefficients (*r*) was interpreted as <0.3 (negligible), 0.3–0.5 (weak), 0.5–0.7 (moderate), 0.7–0.9 (strong), and > 0.9 (very strong) [39].

Statistical significance for main tests was set at *p* ≤ 0.05. In the event of a statistically significant ANOVA outcome, pairwise comparisons were completed post hoc using the Bonferroni correction. In the text, tables’ and figures’ data are reported as mean ± SD.

## 3. Results

### 3.1. Data Distribution

All data were normally distributed (MVC: *W*_(11)_ = 0.821–0.966, *p* = 0.018–0.848; rmsEMG: *W*_(11)_ = 0.699–0.982, *p* = 0.001–0.976; CSE: *W*_(10)_ = 0.684–0.934, *p* = 0.001–0.490), with the exception of MEP/CMEP ratio values under the internal condition of the stimulation session (*W*_(10)_ = 0.628, *p* = 0.0001) and muscle co-activation (% triceps/biceps brachii rmsEMG) under the internal condition of the stimulation session (*W*_(11)_ = 0.639–0.858, *p* = 0.0001–0.054). Thus, all MEP/CMEP ratio and muscle co-activation values were square root transformed using the Transform function in SPSS, resulting in normal distributions (MEP/CMEP: *W*_(10)_ = 0.740–0.879, *p* = 0.003–0.126; co-activation: *W*_(11)_ = 0.745–0.956, *p* = 0.002–0.715).

### 3.2. Peak Force

**Figure 4:**
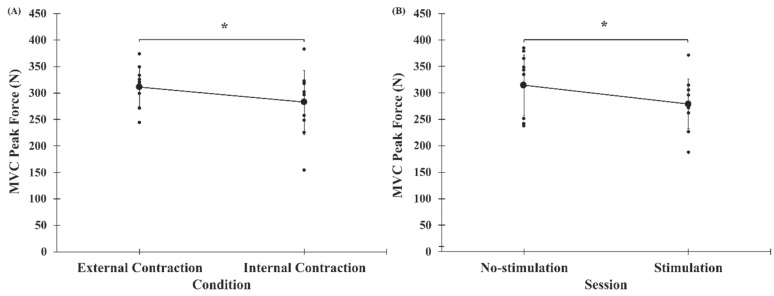
Peak force values for MVCs, measured in Newtons (N). Smaller points represent individual participant data, larger points represent mean, and error bars represent one standard deviation. (**A**) Peak force values for external versus internal conditions, collapsed across all trials (trial 1 to trial 6) and sessions (no-stimulation, stimulation), demonstrating the significant main effect of CONDITION. (**B**) Peak force values for no-stimulation versus stimulation session, collapsed across all trials (trial 1 to trial 6) and conditions (external condition, internal condition), signifying the significant main effect of SESSION. *, statistically significant at *p* < 0.05.

MVC peak force are shown in Figure 4, Table 1 and Table 2. The three-way repeated-measures ANOVA on peak force measurements from elbow flexor MVCs revealed three statistically significant main effects. First, a statistically significant main effect of CONDITION (*F*_(1, 10)_ = 7.033, *p* = 0.024) showed that force was significantly less under the internal conditions (282.4 ± 60.3 N) versus the external condition (310.7 ± 11.3 N) (Figure 4A). Next, a statistically significant main effect of SESSION (*F*_(1, 10)_ = 6.076, *p* = 0.033) demonstrated that force measurements were significantly smaller during the stimulation session (279.0 ± 47.1 N) than the no-stimulation session (314.1 ± 57.5 N) (Figure 4B). Finally, there was a statistically significant main effect of TRIAL (*F*_(5, 50)_ = 14.262, *p* = 0.00001) (see Table 2 for multiple comparisons). Neither the TRIAL × CONDITION (*F*_(5, 50)_ = 1.701, *p* = 0.152), TRIAL × SESSION (*F*_(5, 50)_ = 0.211, *p* = 0.891), CONDITION × SESSION (*F*_(5, 50)_ = 1.365, *p* = 0.270), nor TRIAL × CONDITION × SESSION interactions (*F*_(5, 50)_ = 1.344, *p* = 0.281) were statistically significant.

### 3.3. Electromyography (EMG)

Biceps brachii and triceps brachii rmsEMG data are displayed in Table 1 and Table 2.

#### 3.3.1. Biceps Brachii

No-stimulation session. For biceps brachii rmsEMG during the no-stimulation session there was a statistically significant main effect of TRIAL (*F*_(5, 50)_ = 7.341, *p* = 0.001) (see Table 2 for multiple comparisons). The main effect of CONDITION trended towards significance (*F*_(1, 10)_ = 3.958, *p* = 0.075) and indicated that biceps brachii rmsEMG tended to be greater under the external condition (0.73 ± 0.51% MVC) compared to internal condition (0.60 ± 0.38% MVC). The TRIAL × CONDITION interaction effect was not statistically significant (*F*_(5, 50)_ = 1.83, *p* = 0.133) (Table 2).

Stimulation session. During the stimulation session, the main effect of TRIAL trended towards significance (*F*_(5, 50)_ = 3.317, *p* = 0.068) and suggested that rmsEMG tended to be greater under trial 3 (0.58 ± 0.33% MVC) versus trial 4 (0.53 ± 0.33% MVC) (see Table 2 for multiple comparisons). Otherwise, there was neither a statistically significant main effect of CONDITION (*F*_(1, 10)_ = 2.407, *p* = 0.152) nor TRIAL × CONDITION interaction effect (*F*_(5, 50)_ = 0.506, *p* = 0.565) (Table 2).

#### 3.3.2. Triceps Brachii

No-stimulation session. With reference to triceps brachii rmsEMG throughout the no-stimulation session, there were no statistically significant main effects of TRIAL (*F*_(5, 50)_ = 1.722, *p* = 0.210) or CONDITION (*F*_(1, 10)_ = 2.178, *p* = 0.171), nor a two-way TRIAL × CONDITION interaction effect (*F*_(5, 50)_ = 0.510, *p* = 0.528).

Stimulation session. In the stimulation session, there were no statistically significant effects of TRIAL (*F*_(5, 50)_ = 1.443, *p* = 0.226), CONDITION (*F*_(1, 10)_ = 0.141, *p* = 0.716), or TRIAL × CONDITION (*F*_(5, 50)_ = 0.642, *p* = 0.583), for triceps brachii rmsEMG.

#### 3.3.3. Co-Activation

**Figure 5:**
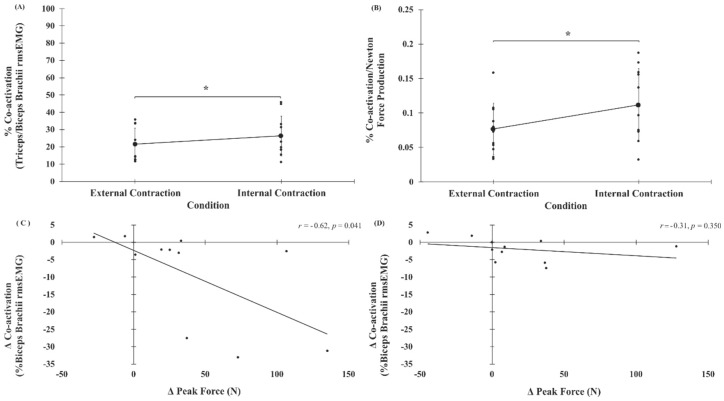
Data expressing the relationship between muscle co-activation and MVC peak force. In panels A–B, smaller points represent individual participant data, larger points represent mean, and error bars represent one standard deviation. In panels D–E, points represent individual data. (**A**) Muscle co-activation values for external and internal conditions, collapsed across trials (trial 1 to trial 6) and sessions (no-stimulation, stimulation), demonstrating the significant main effect of CONDITION. (**B**) Percentage of muscle co-activation/MVC peak force (co-activation per Newton force production) for external versus internal conditions, collapsed across session (no-stimulation, stimulation), illustrating the significant main effect of CONDITION. (C-D) Scatterplots demonstrating relationship between changes in MVC peak force and triceps brachii/biceps brachii co-activation across external and internal conditions during the (**C**) no-stimulation and (**D**) stimulation sessions. *, statistically significant at *p* < 0.05.

Muscle co-activation data (expressed as % triceps/biceps rmsEMG) are shown in Figure 5A and Table 1 and Table 2. The three-way repeated-measures ANOVA on percentage values of co-activation demonstrated a statistically significant main effect of CONDITION (*F*_(1, 10)_ = 8.438, *p* = 0.016), whereby muscle co-activation was significantly greater under the internal condition (26.3 ± 11.5%) versus external contraction condition (21.5 ± 9.4%) (Figure 5A).

The main effects of TRIAL (*F*_(5, 50)_ = 2.123, *p* = 0.136) and SESSION (*F*_(1, 10)_ = 0.029, *p* = 0.869) were not statistically significant. Likewise, neither the TRIAL × CONDITION (*F*_(5, 50)_ = 0.175, *p* = 0.971), TRIAL × SESSION (*F*_(5, 50)_ = 0.419, *p* = 0.833), CONDITION × SESSION (*F*_(5, 50)_ = 1.969, *p* = 0.191), nor TRIAL × CONDITION × SESSION (*F*_(5, 50)_ = 2.072, *p* = 0.144) interaction effects were statistically significant.

### 3.4. Corticospinal Excitability (CSE)

CSE data are presented for each condition (external cue, internal cue), collapsed across trials (trial 1 to trial 6) in Table 3.

There was no statistically significant difference (*t*_(9)_ = −0.508, *p* = 0.624; *t*_(9)_ = 0.598, *p* = 0.565; *t*_(9)_ = 0.340, *p* = 0.742; and *t*_(9)_ = −1.215, *p* = 0.255) in Mmax, MEP, or CMEP amplitudes or MEP/CMEP ratios, respectively across external cued condition and internal cued conditions. A sample of MEP, CMEP, and M-wave responses during stimulation is presented Figure 2.

### 3.5. Co-Activation/MVC Peak Force

There was a statistically significant main effect of CONDITION for ratios of co-activation/Newton force produced in MVCs (*F*_(1, 10)_ = 11.307, *p* = 0.007), which indicated that under the external condition (0.08 ± 0.04%) less muscle co-activation occurred per Newton of force production compared to the internal condition (0.11 ± 0.05%; *p* = 0.007) (Figure 5B). Neither the main effect of SESSION (*F*_(1, 10)_ = 0.131, *p* = 0.725) nor the CONDITION × SESSION two-way interaction effect (*F*_(5, 50)_ = 1.333, *p* = 0.275) reached statistical significance.

### 3.6. Relationship between Change in Peak Force and Co-Activation

Values of percent muscle co-activation per Newton of force production in MVCs, and correlations between changes in MVC peak force and triceps brachii/biceps brachii co-activation, are shown in Figure 5B–D and Table 1 and Table 2.

#### Correlations

***No***-***stimulation session.*** During the no-stimulation session there was a statistically significant negative correlation between changes in MVC peak force (38.8 ± 48.6 N) and triceps brachii/biceps brachii co-activation (−9.2 ± 13.9%) across external and internal conditions (*r*_(9)_ = −0.623, *p* = 0.041, moderate correlation), suggesting increased co-activation was related to reduced MVC force production in the internal condition (Figure 5C).

***Stimulation session.*** In the stimulation session the relationship between changes across external and internal conditions in MVC peak force and triceps brachii/biceps brachii co-activation was not present (*r*_(9)_ = −0.312, *p* = 0.350, weak correlation) (Figure 5D).

## 4. Discussion

The purpose of this study was to examine if consistent superior motor performance observed with an external, compared with an internal focus instruction, is mediated by different corticospinal excitability processes. We observed three key findings. First, consistent with the literature, force production was greater with external focus instructions. Second, the greater force outputs were accompanied with lower co-contraction ratios between the biceps and triceps brachii (measured as rmsEMG Triceps Brachii/rmsEMG Biceps Brachii) under the external focus condition, leading to a more effective contractions strategy. Third, the neuromuscular strategy identified with the EMG patterns did not coincide with a change in corticospinal excitability. This finding likely stems from an interaction between the stimulation techniques for measuring CSE and attentional focus. We speculate that the stimulation negated the effect of an external focused cue. This assumption is supported by the higher forces produced under the external focus condition in the non-stimulation session.

**Maximal elbow flexor force is affected by the type of attentional focus cue.** Participants were able to produce more force when provided an external focus cue (310.7 ± 11.3 N) compared to internal cue (282.4 ± 60.3 N) condition during the non-stimulation session. This is consistent with previous research which showed enhanced force production when given an external cue over no cue and internal focus cues. For example, Marchant et al. (2009) found that during concentric elbow flexion completed at a set speed, an external cue led to a higher peak net torque (102.10 ± 2.42% MVC) than the internal condition (95.33 ± 2.08% MVC) [6]. Halperin et al. (2016) reiterated these results showing that when given an external focus cue during an isometric mid-thigh pull, trained athletes applied 9% more force compared to those that received an internal cue, and 5% more force than control [40]. This supports that external focus cues enhance force output compared to internal ones.

While there was an observed difference in force production between conditions in the non-stimulation session, there were no significant changes in force production between conditions during the stimulation session. This finding is not consistent with previous research as it is well documented that attentional focus alters force production [1,5,6,7,40], which can be accounted for by a number of possible reasons. First, the stimulation may have distracted the participants from focusing on the provided cue. For example, the stimulation may have caused discomfort, led to fear, or attracted interest, all of which could distract participants from the provided instruction. Additionally, the notion that the stimulations are provided at random intervals may channeled their attention to anticipation, amplifying the distraction from the cues. Second, the use of the stimulation techniques disrupted areas of the cortex responsible for attention. It is known that transcranial magnetic stimulation (TMS) can disrupt cortical function. For example, Ashbridge et al. (1997) suggested that TMS disrupts an area in the front parietal lobe responsible for the focal attention necessary for feature binding in a conjunction search task [41]. Another study showed that repetitive TMS of the intraparietal sulcus and the frontal eye fields during an auditory spatial attention task impaired visually cued auditory attention [42]. With each stimulation pulse, it is possible that more than just the cortical area of interest was being stimulated [43], and therefore it is possible that cortical areas involved in attention were unintentionally disrupted. It has been studied that attentional focus potentiate short-term plasticity in the motor cortex through the premotor-to-motor connection, which is why TMS stimulation of the motor cortex disrupts attentional focus [44]. Either of the mentioned reasons, or a combination thereof would confound the effects of attentional focus instructions on force production and explain the differences between sessions.

Moreover, force produced in the stimulation session (279.0 ± 47.1 N) was lower compared to the non-stimulation session (314.1 ± 57.5 N), collapsed across the two instruction conditions. This finding is aligned with Button and Behm [45], who showed that the expectation of an interpolated twitch stimulation reduced voluntary force production by 9.5%. However, to date there appears to be a lack of research showing how stimulation of the nervous system using TMS and TMES influences force production. This result should be replicated and expanded upon in future studies as it implies that the use of stimulations could confound a study involving force production

**Mechanisms underlying changes in elbow flexor maximal force with attentional focus cues.**

**Electromyography.** Our results showed greater co-activation with an internal compared to an external cue. This is consistent with a previous study by Lohse et al. (2011), who reported greater co-contraction between the lateral aspect of the soleus and the tibialis anterior with an internal focus cue during a submaximal plantar flexion task [46]. Greater co-activation of the agonist and antagonist musculature is a possible mechanism underpinning why maximal force production is lower during internal focused cues. Based on the current EMG findings, and aligned with Wulf’s [1] “Constrained action hypothesis”, it appears that force production is impaired with an internal cue due to disruption of natural automatized movement as supported by increases in co-activation by increasing the antagonist and decreasing the agonist muscle activity, whereas the greater EMG pattern of the agonists observed during the external cue could be a result of greater motor unit recruitment and/or rate coding. In contrast to some studies, we did not find a significant difference in EMG activity between focus cues. However, a large, albeit not statistically significant, effect was observed in which greater neuromuscular activity of the biceps brachii was associated with an external focus cue, suggesting that external cue leads to greater motor unit recruitment and/or rate coding.

**Corticospinal Excitability.** Corticospinal tract output can be altered by multiple variables, such as exercise, injury, disuse and disease and potentially attentional cues. As force output is increased, both supraspinal and spinal excitability also increased illustrating that changes in the excitability of cortical neurons and/or spinal motoneurons are occurring [26,27]. Measures of corticospinal excitability, specifically supraspinal and spinal excitability were used during one of the two sessions (stimulation session). This allowed us to determine whether or not the increase in maximal elbow flexor force with an external focus cue was due, in part, to enhanced corticospinal excitability at the supraspinal or spinal level(s) or combination thereof, of the biceps brachii. We expected to see an increase or change in corticospinal excitability at the supraspinal or spinal level(s) of the biceps brachii with an external focus cue as increased central drive is a well-known mechanism underlying increases in force production [26,27,34]. However, we were unable to support this possibility with the current study due to a lack of differences in corticospinal excitability responses of the biceps brachii during elbow flexor MVCs when receiving external versus internal focus cues. There are a couple technical considerations that must be noted in relation to the measurement of corticospinal excitability. First, because MEP [26,47] and CMEP [26] amplitudes are dependent on background EMG during isometric contractions and EMG was different between conditions (i.e., attentional cues), it is possible that the MEPs and CMEPs between conditions may not be comparable. Second, MEP and CMEP amplitudes were matched during a 5% MVC to equal 10%–20% of Mmax. The motor output and activation of cortical neurons and spinal motoneurons required to produce a 5% MVC is different than 100% MVC. Because the same stimulation intensities were used during a 5% and a 100% MVC, we cannot rule out that the corticospinal tract (TMS and TMES) during 100% MVC was not optimally stimulated. Thus, the MEP and CMEP responses may have been suboptimal. In fact, during 100% MVC the MEP and CMEP amplitudes were much higher than 10%–20% of Mmax amplitude. Nonetheless, force and EMG and MEP, CMEP and Mmax amplitudes of the biceps brachii were not significantly different between attentional focus cues. To our knowledge, this appears to be the only study examining corticospinal excitability and attentional focus feedback. In view of the possible reasons raised earlier that can account for the lack of effects under the stimulation condition, it seems like a worthwhile attempt to conceptually replicate the current study with participants who are more experienced with isometric contractions and corticospinal excitability techniques, which may reduce its distracting effects, and thus possibly lead to different results.

## 5. Conclusions

In conclusion, force production during a MVC of the elbow flexors followed the known pattern in which external cue leads to superior performance compared to internal cue. This finding was accompanied by greater co-activation of the triceps brachii and biceps brachii which appear to be an underlying mechanism for this impairment. Interestingly, and in contrast to the non-stimulation sessions, the use of stimulation techniques impaired attention by way of distraction or impairment of certain areas in the brain, which nullified the established effects of attentional focus instruction on maximal force production.

## Figures and Tables

**Table 1:** Mean ± SD of MVC force and electromyographic (EMG) data, collapsed across trials (trial 1 to trial 6), presented for conditions (external cue, internal cue) during both no-stimulation and stimulation sessions.

	No-Stimulation		Stimulation	
	External Cue (Range)	Internal Cue (Range)	External Cue (Range)	Internal Cue (Range)
**MVC Peak Force (N)**	333.5 ± 43.7, (242.6–375.5)	294.8 ± 76.7, (184.2–398.6)	287.9 ± 38.7, (245.3–376.0)	270.1 ± 62.4, (124.3–367.1)
**Biceps Brachii rmsEMG**	0.73 ± 0.51, (0.27–1.88)	0.60 ± 0.38, (0.16–1.47)	0.59 ± 0.33, (0.26–1.41)	0.53 ± 0.25, (0.21–1.04)
**Triceps Brachii rmsEMG**	0.12 ± 0.03, (0.07–0.18)	0.15 ± 0.09, (0.05–0.34)	0.11 ± 0.04, (0.05–0.17)	0.12 ± 0.04, (0.05–0.17)
**Co-activation (% Triceps/Biceps rmsEMG)**	22.2 ± 13.8, (8.6–49.6)	31.5 ± 19.5, (12.1–77.2)	24.3 ± 15.7, (11.3–63.0)	26.8 ± 17.5, (12.7–74.4)
**% Co-activation per Newton Force**	0.06 ± 0.04, (0.03–0.14)	0.12 ± 0.08, (0.03–0.24)	0.09 ± 0.06, (0.03–0.23)	0.11 ± 0.08, (0.03–0.29)

N: Newton, rmsEMG: Root mean square of EMG signal.

**Table 2:** Mean ± SD of MVC force and electromyographic data, collapsed across sessions (no-stimulation, stimulation) and conditions (external condition, internal condition), for MVC trial 1 to trial 6.

		1 (Range)	2 (Range)	3 (Range)	4 (Range)	5 (Range)	6 (Range)
**MVC Peak Force (N)**		313.8 ± 45.8, (229.9–373.8) ^*d*,*e*^	304.7 ± 42.1, (224.4–372.2) ^*d*,*e*^	308.1 ± 36.2, (258.9–377.7) ^*d*,*e*^	276.0 ± 34.1, (227.5–336.1) ^*a*,*b*,*c*^	284.0 ± 30.4, (23.4–320.9) ^*a*,*b*,*c*^	288.6 ± 34.8, (219.4–335.6)
**Biceps Brachii rmsEMG**	**S**	0.72 ± 0.46, (0.25–1.83)	0.70 ± 0.46, (0.22–1.76) *^e^*	0.69 ± 0.45, (0.21–1.71) *^d^*	0.64 ± 0.43, (0.21–1.59) *^c^*	0.64 ± 0.45, (0.23–1.67) *^b^*	0.61 ± 0.40, (0.17–1.50)
	**NS**	0.63 ± 0.28, (0.31–1.10)	0.57 ± 0.31, (0.18–1.27)	0.58 ± 0.33, (0.20–1.38)	0.53 ± 0.33, (0.18–1.35)	0.52 ± 0.26, (0.21–1.14)	0.53 ± 0.27, (0.20–1.15)
**Triceps Brachii rmsEMG (mV·s)**	**S**	0.14 ± 0.05, (0.07–0.23)	0.15 ± 0.06, (0.07–0.26)	0.14 ± 0.06, (0.06–0.25)	0.14 ± 0.08, (0.06–0.35)	0.13 ± 0.06 (0.06–0.25)	0.12 ± 0.05, (0.06–0.20)
	**NS**	0.12 ± 0.04, (0.05–0.16)	0.11 ± 0.04, (0.05–0.17)	0.12 ± 0.04, (0.06–0.17)	0.11 ± 0.04, (0.05–0.18)	0.12 ± 0.05, (0.05–0.19)	0.11 ± 0.04, (0.05–0.16)
**Co-activation (%Triceps/Biceps rmsEMG) (mV·s)**		23.9 ± 9.8, (12.8–40.5)	27.0 ± 13.4, (13.1–53.9)	25.5 ± 12.1, (12.3–48.7)	27.6 ± 12.6, (12.1–55.8)	26.8 ± 12.9, (12.5–54.5)	26.3 ± 14.2, (11.8–55.9)
**%Co-activation per Newton Force**		0.08 ± 0.03, (0.03–0.13)	0.09 ± 0.04, (0.04–0.16)	0.08 ± 0.04, (0.03–0.15)	0.10 ± 0.04, (0.04–0.18)	0.09 ± 0.04, (0.04–0.17)	0.09 ± 0.05, (0.04–0.18)

N: Newton, rmsEMG: Root mean square of EMG signal, S: Stimulation, NS: No stimulation. *^a^*, statistically significant difference versus trial 1, *p* < 0.05. *^b^*, statistically significant difference versus trial 2, *p* < 0.05. *^c^*, statistically significant difference versus trial 3, *p* < 0.05. *^d^*, statistically significant difference versus trial 4, *p* < 0.05. *^e^*, statistically significant difference versus trial 5, *p* < 0.05.

**Table 3:** Mean ± SD of the corticospinal excitability (CSE), collapsed across trials (trial 1 to trial 6), for external and internal contraction conditions. Data presented as mean (M), standard deviation (SD), and range.

	External Cue (Range)	Internal Cue (Range)
**Mmax Amplitude (mV)**	8.62 ± 4.97, (3.0–20.1)	8.48 ± 5.0, (1.63–17.47)
**MEP Amplitude (Ratio of Mmax)**	0.83 ± 0.36, (0.48–1.65)	1.01 ± 0.78, (0.48–2.95)
**CMEP Amplitude (Ratio of Mmax)**	0.64 ± 0.38, (0.30–1.76)	0.79 ± 0.71, (0.23–2.20)
**MEP/CMEP Ratio**	1.64 ± 0.99, (0.92–3.64)	1.97 ± 1.77, (0.82–6.44)

Mmax: Maximal compound motor unit action potential, MEP: motor evoked potential, CMEP: Cervicomedullary MEP.
